# Effect of octreotide on oxidative stress in the erythrocyte and kidney tissue in adriamycin-induced experimental nephrotic syndrome model

**DOI:** 10.1590/2175-8239-JBN-2022-0180en

**Published:** 2023-07-31

**Authors:** Sibel Cavdar, Alev Garip Acar, Asuman Camyar, Ender Hür, Eser Yıldırım Sozmen, Sait Sen, Melih Ozısık, Yasemin Delen Akcay, Elif Duman, Sena Gönen, Fehmi Akcicek, Soner Duman

**Affiliations:** 1 Ege University, Medical Faculty Hospital, Department of Internal Medicine, Izmir, Turkey.; 2 Izmir Atatürk Training and Research Hospital, Department of Internal Medicine, Izmir, Turkey.; 3 Izmir Çiğli Training and Research Hospital, Department of Internal Medicine, Izmir, Turkey.; 4 Uşak University Medical Faculty Hospital, Department of Internal Medicine, Usak, Turkey.; 5 Ege University Medical Faculty Hospital, Department of Medical Biochemistry, Izmir, Turkey.; 6 Ege University Medical Faculty Hospital, Department of Pathology, Izmir, Turkey.; 7 Izmir Tepecik Training and Research Hospital, Department of Internal Medicine, Izmir, Turkey.; 8 Suat Seren Chest Diseases and Surgery Training and Research Hospital, Izmir, Turkey.; 9 Ege University Medical Faculty Hospital, Department of Microbiology, Izmir, Turkey.

**Keywords:** Nephrotic Syndrome, Octreotide, Reactive Oxygen Species, Catalase, Thiobarbituric Acid Reactive Substance, Oxidative Stress, Síndrome Nefrótica;, Octreotide, Espécies Reativas de Oxigênio, Catalase, Substância Reativa ao Ácido Tiobarbitúrico, Estresse Oxidativo

## Abstract

**Introduction:**

Nephrotic syndrome (NS) is one of the reasons of end-stage kidney disease, and elucidating the pathogenesis and offer new treatment options is important. Oxidative stress might trigger pathogenesis systemically or isolated in the kidneys. Octreotide (OCT) has beneficial antioxidant effects. We aimed to investigate the source of oxidative stress and the effect of OCT on experimental NS model.

**Methods:**

Twenty-four non-uremic Wistar albino rats were divided into 3 groups. Control group, 2 mL saline intramuscular (im); NS group, adriamycin 5 mg/kg intravenous (iv); NS treatment group, adriamycin 5 mg/kg (iv) and OCT 200 mcg/kg (im) were administered at baseline (Day 0). At the end of 21 days, creatinine and protein levels were measured in 24-hour urine samples. Erythrocyte and renal catalase (CAT) and thiobarbituric acid reactive substance (TBARS) were measured. Renal histology was also evaluated.

**Results:**

There was no significant difference among the 3 groups in terms of CAT and TBARS in erythrocytes. Renal CAT level was lowest in NS group, and significantly lower than the control group. In treatment group, CAT level significantly increased compared with NS group. In terms of renal histology, tubular and interstitial evaluations were similar in all groups. Glomerular score was significantly higher in NS group compared with control group and it was significantly decreased in treatment group compared to NS group.

**Conclusions:**

Oxidative stress in NS might be due to the decrease in antioxidant protection mechanism in kidney. Octreotide improves antioxidant levels and histology in renal tissue and might be a treatment option.

## Resumo

**Introdução:**

Síndrome nefrótica (SN) é uma das causas de doença renal em estágio terminal. É importante elucidar a patogênese e oferecer novas opções de tratamento. Estresse oxidativo pode desencadear a patogênese sistemicamente ou isoladamente nos rins. O octreotide (OCT) tem efeitos antioxidantes benéficos. Nosso objetivo foi investigar a fonte de estresse oxidativo e efeito do OCT no modelo experimental de SN.

**Métodos:**

Dividimos 24 ratos albinos Wistar não urêmicos em 3 grupos. Grupo controle, 2 mL de solução salina intramuscular (im); grupo SN, adriamicina 5 mg/kg intravenosa (iv); grupo tratamento SN, adriamicina 5 mg/kg (iv) e OCT 200 mcg/kg (im) foram administrados no início do estudo (Dia 0). Aos 21 dias, mediram-se os níveis de creatinina e proteína em amostras de urina de 24 horas. Mediu-se a catalase (CAT) eritrocitária e renal e a substância reativa ao ácido tiobarbitúrico (TBARS). Avaliou-se também histologia renal.

**Resultados:**

Não houve diferença significativa entre os três grupos em termos de CAT e TBARS em eritrócitos. O nível de CAT renal foi menor no grupo SN e significativamente menor que no grupo controle. No grupo tratamento, o nível de CAT aumentou significativamente em comparação com o grupo SN. Quanto à histologia renal, as avaliações tubular e intersticial foram semelhantes em todos os grupos. O escore glomerular foi significativamente maior no grupo SN em comparação com o grupo controle e diminuiu significativamente no grupo de tratamento em comparação com o grupo SN.

**Conclusões:**

Estresse oxidativo na SN pode ser devido à diminuição do mecanismo de proteção antioxidante nos rins. O octreotide melhora níveis de antioxidantes e histologia do tecido renal e pode ser uma opção de tratamento.

## Introduction

Nephrotic syndrome (NS) is one of the causes of end stage kidney disease, and the pathophysiological mechanisms are important for new treatment options^1^. Although immunological mechanisms, autoimmunity, and genetic predisposition play a role in the pathogenesis of NS, it might also develop as a result of oxidative stress, which is an imbalance between reactive oxygen species (ROS) production and antioxidant defense mechanisms^2^. ROS is thought to play an important role also in the pathogenesis of proteinuria by causing increased glomerular wall permeability and podocyte migration^3,4^.

It is possible to produce NS experimentally with different methods to study the pathophysiology of NS^5^. Adriamycin (generic name is doxorubicin (DOX)) is an anthracycline group antineoplastic agent that induces nephropathy experimentaly^6,7^. In the adriamycin-induced nephrotic syndrome model, adriamycin stimulates oxidative damage in the glomeruli, increases podocyte damage, causes glomerular basement membrane changes, and creates minimal change disease**/**focal segmental glomerulosclerosis-like damage^8,9^. An adriamycin-induced nephropathy model is induced by a single tail vein injection of 5–7.5 mg/kg adriamycin^10^. After intravenous (iv) administration, adriamycin is rapidly cleared from plasma and accumulates in tissues, mainly in the kidney^11^. For this reason, its nephrotoxic feature is evident. Adriamycin causes severe glomerulosclerosis, interstitial fibrosis and inflammation, glomerular endothelial cell and podocyte injury^12^. In addition, it has been suggested that oxidative stress is responsible for the pathogenesis of proteinuria in this model^13^.

In humans, oxidant products are constantly formed as a result of normal aerobic metabolism^14^. Under normal physiological conditions, oxidant production is balanced by antioxidant mechanisms, thus preventing oxidative damage^15^. Under stress, the balance between ROS and the antioxidant system is disrupted in favor of ROS, resulting in oxidative stress and cytotoxicity^16,17^. Oxidative stress causes cell damage by lipid peroxidation, protein oxidation, deoxyribonucleic acid (DNA) mutations and breaks, cytotoxic effects, and disruptions in signaling^17^.

Somatostatin is a general inhibitory tetradecapeptide neurohormone that has various immunomodulatory and anti-inflammatory effects^18,19^. Octreotide (OCT), a synthetic analogue of somatostatin, has an octapeptide structure and is resistant to metabolic degradation^20^. In addition, its duration of action is longer than the natural hormone^20^. The antioxidant properties of OCT have also been reported in some clinical studies and various experimental models^21,22^.

In the present study, the aim was to investigate the source of oxidative stress and whether OCT is a useful therapeutic agent for adriamycin-induced NS model in rats through free oxygen radicals.

## Methods

### Study Protocol

Twenty-four nonuremic Wistar albino male rats (n = 24; weight 180–220 gram) obtained from Ege University Laboratory Animals Application and Research Center (Izmir, Türkiye) were randomly divided into three equal groups. They were housed in polycarbonate cages under 24°C room temperature with a 12-hour light/dark cycle and fed a standard laboratory diet (40 g/day) and had free access to tap water. The Animal Ethics Committee of Ege University Hospital approved the study design (Ethical Approval Number: 2010–33). The institutional and national guidelines for the care and use of laboratory animals were followed.

#### Treatments

All injections were administered at the beginning of the study (Day 0). No other injections were given to any group in the following days until the day of sacrifice. The treatments and procedures applied are summarized in Table 1. The groups were formed as follows.

**Table 1.** Treatments and procedures used to the groups

	Injection substances	Day of injections	Day of placement of rats in metabolic cages	Day of sacrifice, injection of ketamine, HCL anesthesia
Group 1:	2 mL saline (im)	0	20	21
Control group				
(n = 8)				
Group 2:	DOX 5mg/kg (iv)	0	20	21
NS group				
(n = 8)				
Group 3:	DOX 5mg/kg (iv)	0	20	21
NST group				
(n = 8)	OCT 200mcg/kg (im)			

NS: nephrotic syndrome; NST: nephrotic syndrome + treatment; DOX: doxorubicin; OCT: octreotide.

1) Group 1: Control group (n = 8): 2 mL saline was administered intramuscularly (im);
2) Group 2: Nephrotic syndrome (NS) group (n = 8): 5 mg/kg adriamycin (Doxorubicin Hexal^®^; Sandoz, Basel, Switzerland) was administered (iv via tail vein);
3) Group 3: Nephrotic syndrome treatment (NST) group (NS + OCT) (n = 8): 5 mg/kg adriamycin (iv via tail vein) and 200 mcg/kg octreotide (Sandostatin LAR^®^; Novartis, Basel, Switzerland) was administered (im).

On the 20^th^ day after injections, all rats were placed in metabolic cages for collection of 24-hour urine. Metabolic cages allow separate collection of urine and feces of experimental animals. On the 21^st^ day, rats were anesthetized with intraperitoneal injection of ketamine HCL (Ketalar®; Pfizer, Istanbul, Türkiye) anesthesia (60 mL/kg body weight) and blood samples were immediately collected through direct cardiac puncture in sacrificed rats. Semiquantitative assessment of kidneys was carried out by the same pathologist, who was unaware of the groups.

### Functional Parameters

Serum levels of total protein, total cholesterol, triglyceride, and creatinine were measured spectrophotometrically with commercial kits (Biolabo Reagents, Maizy, France).

Total urinary protein concentration (milligrams per deciliters) was determined using the Lowry method.

### Determination of Oxidative Stress

The ROS levels in biological samples can be measured directly or by the assessment of oxidative damage and antioxidant status. Examination of the relevant protein, lipid, and DNA damage can be used indirectly to estimate ROS levels^15^.

Lipids are highly sensitive to oxidant attack. Malondialdehyde (MDA) is one of the main biomarkers for lipid peroxidation assessment, and lipid peroxidation products such as the thiobarbituric acid reactive substance (TBARS) is a commonly used method for its detection^23^.

Antioxidants are examined as two groups, enzymatic and non-enzymatic. Catalase (CAT) is one of the enzymatic antioxidants^15^, and it was selected for our study, since glomerular diseases were shown to increase hydrogen peroxide (H_2_O_2_) in the kidney and the key enzyme in H_2_O_2_ metabolism is CAT^15,24^.

TBARS and CAT levels were measured in kidney tissue and plasma erythrocytes.

#### Erythrocyte

Preparation of hemolysate: after plasma separation, erythrocytes were washed 2 times with 9 g/L NaCl (sodium chloride) solution and hemolyzed by applying ice water (1/5, v/v). CAT activity in the hemolysate was immediately studied. TBARS levels were studied after diluting the hemolysate in physiological saline. After dilution, thiobarbituric acid (TBA) (Sigma-Aldrich, Darmstadt, Germany) was added and boiled at 95 degrees for 30 minutes.

#### Kidney tissue

Tissues were weighed and homogenized with phosphate buffer (1/10: w/v) on ice. Analyses were made after centrifugation at 2000 rpm for 10 minutes.

#### Malondialdehyde (MDA) Measurement

TBA (Sigma-Aldrich, Darmstadt, Germany) was added to the homogenate. After boiling for 20 minutes at 100ºC and centrifuging at 2000 rpm for 10 minutes, colorimetric measurements were made in the supernatant at a wavelength of 532 nm. MDA (nmol/mL) was calculated from the standard graph (1.1.1.3 tetraetoksipropan, Sigma-Aldrich, Darmstadt, Germany). Results were given as nmol/gHb.

#### Catalase measurement

Homogenates were diluted 1:10 with phosphate buffer (50 mM, pH = 7) and catalase activities were determined by ultraviolet (UV) spectrophotometric method based on the degradation of hydrogen peroxide by catalase^25^. A sample was added to a freshly prepared phosphate buffer solution containing 30 mM hydrogen peroxide **(**H_2_O_2_, Carlo Erba, Val de Reuil, France). The decrease in absorbance at 240 nm wavelength was read for 2 minutes at 15-second intervals. The k value and enzyme amount were calculated by finding the most suitable absorbances for each analysis according to linear regressions.

### Structural Parameters

For histopathological evaluation, the capsule was stripped from the kidneys, which were then divided transversely. The kidneys were left in approximately 4% formalin solution, then processed for paraffin embedding. Paraffin sections of 3–5 mm in thickness were cut from the blocks, routinely prepared, stained with hematoxylin and eosin, and then evaluated under a light microscope (BX, Olympus, Tokyo, Japan) by a single pathologist blinded to the groups. Glomerular sclerosis, tubular necrosis, and interstitial inflammation were evaluated semi quantitatively from 0 to 4. Histopathological scoring is summarized in Table 2.

**Table 2.** Histopathological scores

Scores for histopathological evaluation	Glomerular sclerosis (GS)	Interstitial inflammation II)	Tubular necrosis (TN)
0	Ordinary. Absence of GS.	Ordinary. Absence of II.	Ordinary. Absence of TN.
1	Suspicion of glomerular adhesion, sclerosis could not be determined clearly. The Bowman distance is narrow and the glomerular ball touches the capsule.	Inflammatory infiltration in a microscopic field in the cortical area.	Tubular vacuolar changes.
2	Less than 5% segmental sclerosis and/or adhesion to Bowman’s capsule. Affects less than 5% of the glomeruli and only less than 10% of the glomerulus.	More pronounced inflammatory infiltration in the cortical area, focal, not exceeding 10%.	Tubular regenerative features – tubular hyperchromasia and change in chromatin pattern.
3	GS: 10%−25%	II: 10−25%	Tubular degeneration/regeneration as well as tubules casts together.
4	GS > 25%	II > 25%	Diffuse tubular necrosis.

GS: Glomerular sclerosis; II: Interstitial inflammation; TN: Tubular necrosis.

#### Glomerular sclerosis (GS), defined as adhesions, sclerosis and proliferation affecting bowman distance and glomeruli

Scoring: Glomeruli

0. Ordinary, absence of GS.
1. Suspicion of glomerular adhesion and sclerosis could not be determined clearly. The Bowman distance is narrow, and the glomerular ball touches the capsule.
2. Less than 5% segmental sclerosis and/or adhesion to Bowman’s capsule. Affects less than 5% of the glomeruli and less than 10% of the glomerulus.
3. More than 10% glomerular sclerosis and adhesions.
4. More than 25% glomerular sclerosis and adhesions.

#### Interstitial inflammation (II), defined as infiltration of inflammatory cells in perivascular and interstitial areas

Scoring: Interstitium

0. Ordinary, absence of II.
1. Inflammatory infiltration in a microscopic field in the cortical area.
2. More pronounced inflammatory infiltration in the cortical area, focal, not exceeding 10%.
3. 10–25% inflammatory infiltration.
4. More than 25% inflammatory infiltration.

#### Tubular necrosis (TN), defined as loss of epithelial cells of the nucleus, dark acidophilic cytoplasm, loss of tubular epithelial cells into tubular lumen, and acellular sections of tubules

Scoring: Tubules

0. Ordinary, absence of TN.
1. Tubular vacuolar changes.
2. Tubular regenerative features, tubular hyperchromasia and change in chromatin pattern.
3. Tubular degeneration and regeneration as well as tubular casts together.
4. Diffuse tubular necrosis.

### Statistical Analysis

Statistical analysis was performed using the SPSS 22.0 program (IBM Corp., Armonk, NY, USA). Continuous variables are reported as means ± standard deviations (SD). Nonparametric tests Kruskal Wallis and Mann-Whitney U were used to compare independent group differences, as the parametric test assumptions were not met. Kruskal Wallis test was applied for comparing the means of the three groups. Mann-Whitney U test was used to compare binary groups. A p < 0.05 was considered as significant.

## Results

Urine protein excretion was higher in the NS group compared to both the control group (p < 0.05) and the NST group (p < 0.05). Serum total protein levels were lower in the NS group compared with the control group and with the NST group (p < 0.05, p < 0.05). There was no statistically significant difference in plasma creatinine values in the NS group and NS treatment group compared to the control group. Creatinine levels were similar in all 3 groups. Although the serum triglyceride value in the NS group was higher than the control group (p < 0.05), no significant difference was observed between NST and NS groups (p > 0.05). There was also no significant difference among groups in terms of serum total cholesterol values (p > 0.05). Plasma and urine biochemical measurements of the groups are shown in Table 3 and Graphic 1.

**Table 3.** Clinical, laboratory and histological findings

		Control group.(n = 8)	Nephrotic Syndrome (NS) Group. (n=8)	Nephrotic Syndrome Treatment (NST) Group. (n = 8)
		mean ± SD	mean ± SD	mean ± SD
**Rat**	Weight (gr)	217 ± 17	235 ± 12	209 ± 9
**Urine**	Volume (cc)	3.8 ± 0.3	8.5 ± 1a	6 ± 1
	Proteinuria (mg/dL/day)	12.7 ± 0.4	227 ± 60a	52 ± 20b
**Plasma**	Creatinine (mg/dL)	0.6 ± 0.06	0.4 ± 0.07a	0.4 ± 0.04a
	Total protein (g/dL)	5.8 ± 0.02	3.7 ± 0.6a	5.9 ± 0.05b
	Cholesterol (mg/dL)	188 ± 5	248 ± 19	222 ± 28
	Triglyceride (mg/dL)	91 ± 5	138 ± 13a	135 ± 24
**Erythrocyte**	CAT (U/gHb)	2464 ± 500	2547 ± 660	1442 ± 556
	TBARS(nmol/gHb)	116 ± 14	95 ± 6.3	105 ± 8
**Kidney Tissue**	CAT (U/mL)	53 ± 2	35 ± 4.3a	50 ± 2.47b
	TBARS (mmol/mg)	0.35 ± 0.03	0.38 ± 0.02	0.36 ± 0.01
**Kidney Histology**	Glomerular	0 ± 0	1.1 ± 0.35a	0 ± 0b
	Interstitium	0.6 ± 0.4	0.2 ± 0.2	0.3 ± 0.2
	Tubular	0.34 ± 0.3	0.50 ± 0.27	1.3 ± 0.4

Numerical values are given as mean ± SD and Kruskal Wallis test was used for mean comparison of the three groups. Mann-Whitney U test was used to compare the means of two independent groups.

CAT: catalase; TBARS: thiobarbituric acid reactive substance; SD: standard deviation.

a: versus control; b: versus nephrotic syndrome group; (p < 0.05).

**Graphic 1.**
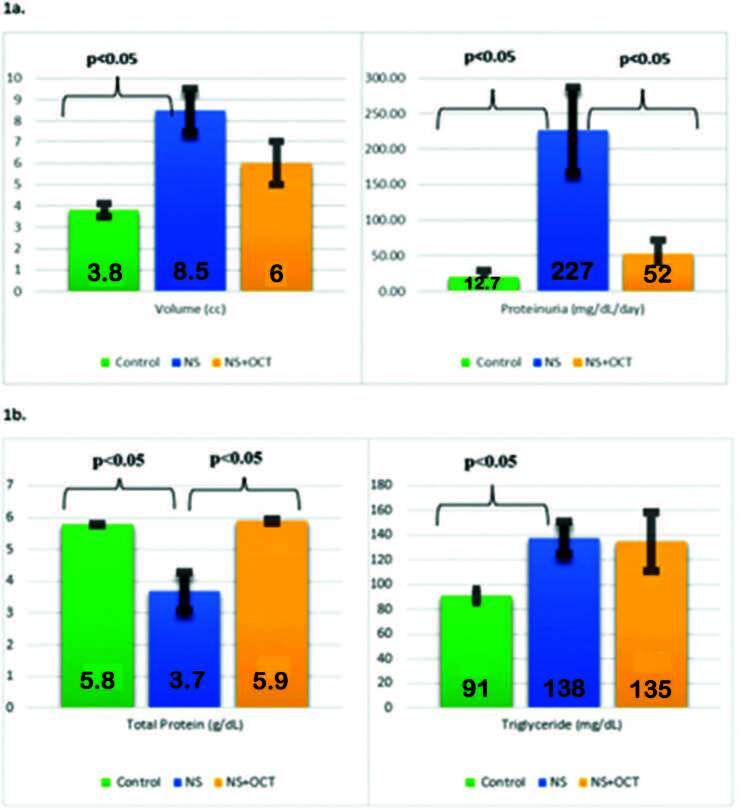
Development of nephrotic syndrome. 1a: Change of urine volume and proteinuria; 1b: Change of plasma total protein and triglyceride. Nephrotic syndrome development was shown by changes in urine volume, proteinuria, plasma total protein, and triglyceride levels. NS: Nephrotic syndrome; OCT: Octreotide.

**Figure 1.**
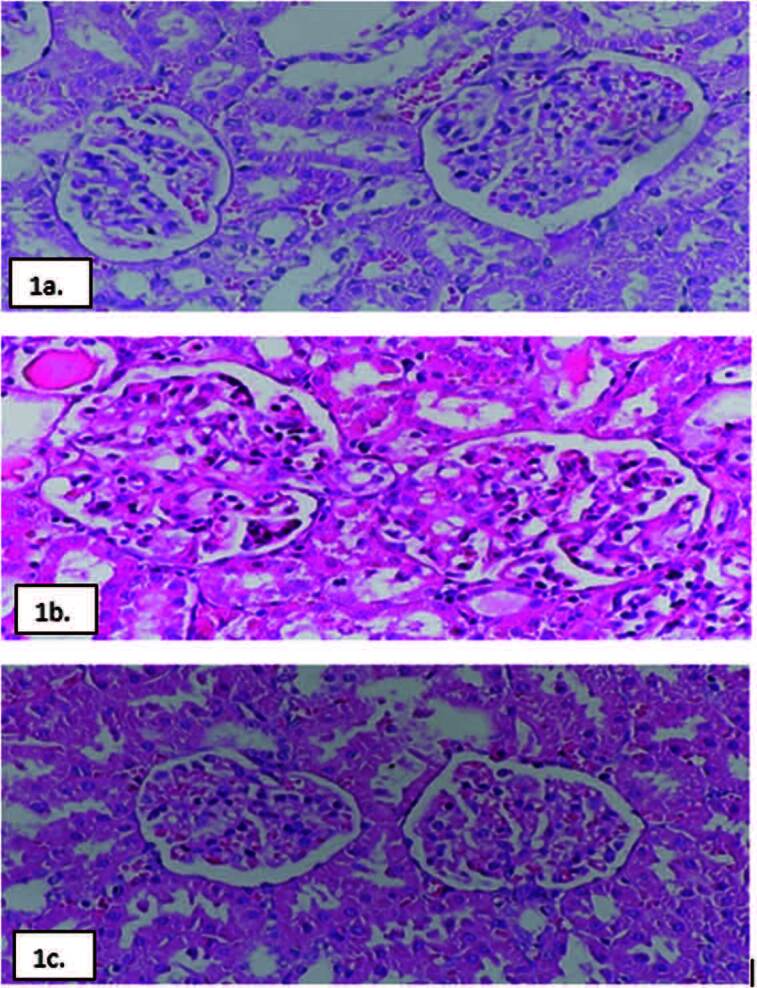
Renal Pathology. **1a.** Control group, **1b.** NS group, **1c**. NST (NS + OCT) group. Light microscopic view of kidneys of the control, NS, and NST (NS + OCT) groups. (H&E, X40). NS: Nephrotic syndrome; NST: Nephrotic syndrome + treatment; OCT: Octreotide; H&E; Hematoxylin and eosin. **1a.** Normal histology is observed in the control group. **1b**. In the NS group, adhesion, sclerosis, and accompanying proliferation are observed in Bowman’s capsule. **1c**. In the NST (NS + OCT) group, Bowman distance is observed as normal, sclerosis and proliferation are not observed.

There was no significant difference in erythrocyte CAT and TBARS levels among the 3 groups (Table 3, Graphic 2). In kidney tissue, TBARS levels were increased in the NS group compared to the control group (p > 0.05) and decreased in the treatment group compared to the NS group (p > 0.05). However, this difference was not statistically significant (Table 3, Graphic 3). Kidney tissue catalase level was decreased in the NS group compared to the control group (p < 0.05), and a statistically significant increase was found in the treatment group compared to the NS group (p < 0.05) (Table 3, Graphic 3). In the histological evaluation of the kidney, no significant difference was found among the 3 groups in tubule and interstitial structures (p > 0.05 for all comparisons). Glomerular pathology score increased in the NS group compared to the control group (p < 0.05) and decreased significantly in the treatment group compared to the NS group (Figure 1, Table 3, Graphic 4).

**Graphic 2.**
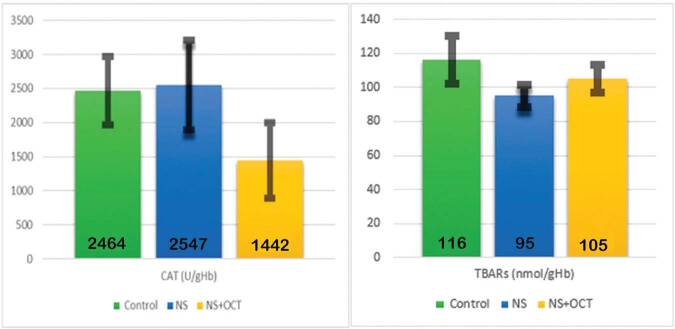
Changes in CAT and TBARS in erythrocyte. There are no significant changes in CAT and TBARS in erythrocyte the control group, NS group, and NS + OCT group (p > 0.05). CAT: catalase; TBARS: thiobarbituric acid reactive substance; NS: Nephrotic syndrome; OCT: octreotide.

**Graphic 3.**
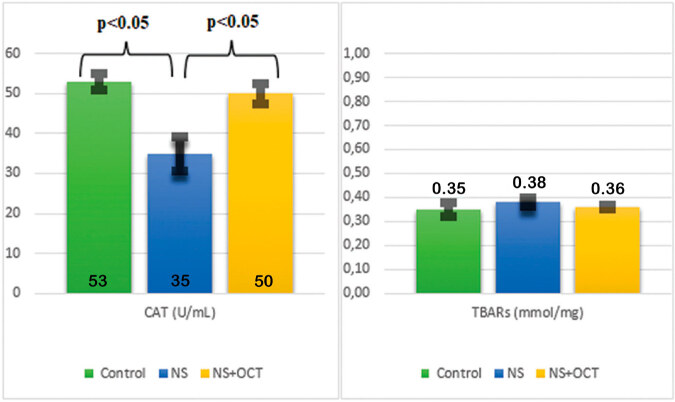
Changes of CAT and TBARS in kidney tissue. CAT: catalase; TBARS: thiobarbituric acid reactive substance; NS: Nephrotic syndrome; OCT: octreotide.

**Graphic 4.**
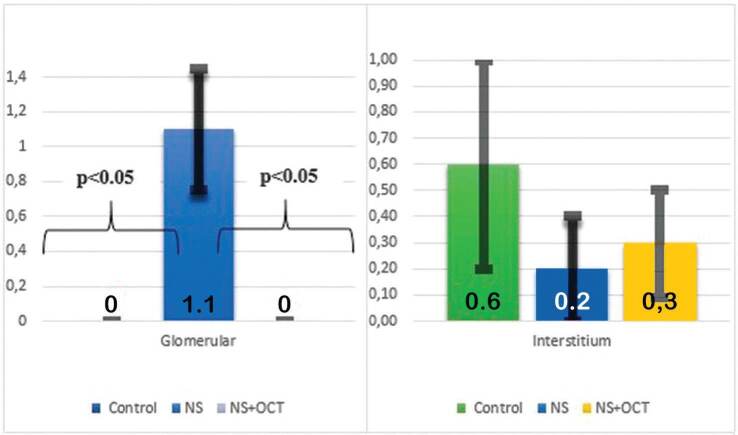
Effect of OCT on kidney histology. p < 0.05 for glomeruli, p > 0.05 for interstitium. NS: Nephrotic syndrome; OCT: octreotide.

## Discussion

Nephrotic syndrome etiologically encompasses a variety of diseases and is characterized by proteinuria, hypoalbuminemia, hyperlipidemia, and edema^1^. Proteinuria is an important risk factor for chronic renal failure progression and there is a strong relationship between the urine protein level and oxidative stress in the kidney^26,27^. Moreover, NS is an important cause of end-stage kidney disease^1^. For this reason, it is extremely important to elucidate the pathogenesis of NS and proteinuria for developing new treatment options. Therefore, experimental NS models are being developed, and studies on this subject continue. In this study, 3 weeks after administering a single 5-mg/kg adriamycin dose iv, NS developed with increased proteinuria in the 24-hour urine, decreased serum total protein, and high glomerular sclerosis score in the histological evaluation.

Although the underlying etiologies and pathogenesis of NS are dissimilar, ROS might play an important role in the etiopathogenesis of proteinuria^27,28,29^. ROS formation is triggered in most of kidney diseases, including NS^27^. Although this suggests that ROS formation is the result of NS, other animal experiments have also shown that ROS causes NS by affecting podocytes^27,29^, which are highly vulnerable to oxidative damage^3^. ROS may be involved in the pathogenesis of glomerular injury by toxic, ischemic, and immunologic mechanisms^30^. Increased levels of ROS within cells can lead to random and irreversible oxidation events, causing permanent damage to macromolecules, such as DNA, lipids, and proteins, ultimately contributing to cell death and/or development of diseases^14^. Glomerular injury is directly mediated by increased generation of ROS, such as H_2_O_2_, OH¯, superoxide anion radicals, and lipid peroxidation products^3^.

It is unclear whether the source of oxidative stress in NS pathogenesis is at the systemic or renal level. Some studies have shown that the imbalance between ROS and the antioxidant system in NS is related to the oxidative reaction originating from circulating neutrophils^29,31^. Furthermore, chronic accumulation of advanced oxidation protein products (AOPPs) in plasma has been associated with podocyte loss, proteinuria, and glomerulosclerosis^3^. On the other hand, in a study conducted in children with NS in which oxidative stress was evaluated, no significant difference was found between the remission and control groups in terms of erythrocyte MDA levels^32^. In our study, when the NS group, NS treatment group, and the control group were compared, no significant change was observed in the TBARS and CAT levels measured in erythrocytes. This finding can indicate that adriamycin-induced NS is related to isolated kidney pathology rather than a systemic oxidative reaction. Baud et al.^30^ also reported that in glomerular diseases macrophages isolated from glomeruli produce more radicals than monocytes in peripheral blood. This supports that the impaired balance of the ROS-antioxidant system in NS results from the kidney tissue rather than a systemic oxidative reaction.

Oxidative stress in kidney disease is linked to both antioxidant depletion and increased ROS production^33^. In our study, TBARS levels in kidney tissue were increased in the NS group compared to the control group and decreased in the treatment group compared to the NS group, but this differences were not statistically significant. This might be related with the short half-life of MDA and other lipid peroxidation products^29,34^. However, the TBARS assay is usually considered a good indicator of the overall levels of oxidative stress in a biological material^35^. Besides, it is known that protein oxidation products, which have a longer half-life, are associated with podocyte damage, proteinuria, and the development of focal segmental glomerulosclerosis as well as tubulointerstitial fibrosis^3^.

Studies for new treatment options for NS are ongoing, and studies on ROS, which are thought to play a role in the etiopathogenesis of NS and proteinuria, might be a wise step. Avoiding factors that cause oxidative stress is very important in preventing the formation and progression of many diseases^36^. Because of the involvement of oxidative stress in kidney fibrosis, therapies targeting oxidative stress are promising^27^. As far as we can tell from the literature, OCT, which has been tried in various diseases and shown to influence ROS^22,37^, has never been tried in NS.

Clinical studies have reported antioxidant, anti-inflammatory, immunomodulatory and antiapoptotic properties of OCT and its general inhibitory effect^38^. In the study conducted by Niedermühlbichler and Wiedermann^39^, somatostatin-related peptides had a regulatory role in the metabolism of oxygen radicals. We thought that OCT could be a treatment option in NS with its anti-inflammatory, antifibrotic, antioxidant, and general inhibitory effects. In our study, CAT level in the kidney tissue was significantly lower in the NS group compared to the control group and significantly increased in the treatment group compared to the NS group. In other words, the antioxidant capacity of the NS group decreased compared to the control group and after the OCT application, the antioxidant capacity increased and reached the level of the control group. The histopathological evaluations also supported this data. Glomerular sclerosis score increased in the NS group compared to the control group and decreased in the NS treatment group compared to the NS group, indicating that OCT treatment improved both the deteriorated antioxidant capacity and the histopathology parameters in the kidney tissue.

Proteinuria damages the glomerulus and the tubulointerstitium. Oxidative stress has an important role in tubulointerstitial fibrosis by the activation of the myofibroblast and in glomerulosclerosis by mesangial sclerosis, podocyte abnormality, and parietal epithelial cell injury^27^. Tubulointerstitial damage is common in glomerular diseases and correlates with the degree of proteinuria and renal function^40^. Adriamycin-induced nephropathy creates podocyte injury followed by glomerulosclerosis, tubulointerstitial inflammation, and fibrosis^7^. No difference was observed in tubulointerstitial structures in all 3 groups, and this might be related to the duration of our study. In the literature, tubulointerstitial changes were usually seen at weeks 4 to 6^7^. The 3-week duration of our study might not have been enough to damage the tubules.

The results of this study suggest that impaired mechanisms of antioxidant protection rather than increased ROS production in the kidney play a role in the etiopathogenesis of NS. OCT might be a treatment option by improving antioxidant capacity and histopathological structures in the kidneys.

## Conclusion

 Oxidative stress has a role in the pathogenesis of NS. Our findings were consistent with many studies in the literature. Oxidative stress in NS might be due to impairment of the antioxidant protection mechanism in the kidney. The antioxidant and antifibrotic effect of octreotide, which has been shown in many studies in the literature, was also supported by our study. We have shown for the first time that OCT may improve the decreased antioxidant capacity and the glomerular sclerosis in kidney tissue.

## Limitations of the Study

Whether OCT has an effect on survival is not known. For this reason, a longer study should be planned in the future. If protein oxidation products and ROS like H_2_O_2_ had been measured together with MDA, the change in free radicals in NS could have been clearer. The effects of OCT in antioxidant parameters such as vitamin E, vitamin C, glutathione, glutathione peroxidase, and superoxide dismutase were not assessed, which could more broadly demonstrate the total antioxidant capacity. In addition, there was no OCT group to examine the effect of OCT alone. When planning future studies, these deficiencies should be taken into account so that expanded and improved studies can be performed.
